# Implications of the simple chemical structure of the odorant molecules interacting with the olfactory receptor 1A1

**DOI:** 10.5808/gi.21033

**Published:** 2021-06-30

**Authors:** S. June Oh

**Affiliations:** Department of Pharmacology, Inje University College of Medicine, Busan 47392, Korea

**Keywords:** electron transfer, homology modeling, molecular docking, olfactory receptor

## Abstract

G protein‒coupled receptors (GPCRs), including olfactory receptors, account for the largest group of genes in the human genome and occupy a very important position in signaling systems. Although olfactory receptors, which belong to the broader category of GPCRs, play an important role in monitoring the organism’s surroundings, their actual three-dimensional structure has not yet been determined. Therefore, the specific details of the molecular interactions between the receptor and the ligand remain unclear. In this report, the interactions between human olfactory receptor 1A1 and its odorant molecules were simulated using computational methods, and we explored how the chemically simple odorant molecules activate the olfactory receptor.

## Introduction

It is very important for animals to be able to perceive their surroundings accurately, and this ability is directly linked to survival. Sight and smell are among the senses involved in the initial recognition of external signals by an animal's biological system, and G protein-coupled receptors (GPCRs) are responsible for these pivotal functions.

Since the first GPCR structure with a good resolution (2.8 Å) was reported in 2000 [1], more than 400 GPCR entries produced by methods such as X-ray crystallography or cryogenic electron microscopy have been registered in the RCSB Protein Data Bank (PDB) [2]. Many of the three-dimensional (3D) structures of these seven-transmembrane receptors (7TMRs), including visual receptors, have been revealed through the methods described above; however, the 3D structure of olfactory receptors (ORs) has not yet been reported. Accordingly, the actual binding mode of ORs and their ligands has not been known. This gap in scientific knowledge has hampered research on the actual activation mechanism of ORs by odorant molecules.

It is known that ORs are widely expressed in the olfactory organs, where they carry out their main functions; however, recent reports have suggested that ORs also appear to be active throughout the animal body [3,4]. In addition to discovering the important functions and roles of ORs, it is essential to determine and understand the actual activation mechanisms of functional proteins, including 7TMRs. Signal transduction in 7TMR systems is known to be mediated by structural changes in receptors triggered by ligands. Therefore, it is of the utmost importance to investigate the initial interactions between ligands and receptors.

The simpler the chemical structure of a ligand, the more straightforward it is to characterize the binding and action relationship between the membrane receptor and the ligand through their interactions. In recent research, human olfactory receptor 1A1 (OR1A1) activation was observed in 11 different tumor types by the analysis of single-cell transcriptomes [5]. The molecular weight of the ligands binding to OR1A1 was around 140 Da, which is relatively small, and the ligands have simple structures as biomolecules. The present study was designed to establish a homology model of OR1A1 and to determine the functional group involved in the interaction between the model and the ligand from the binding mode by a computational simulation.

## Methods

### Dataset for OR1A1 ligands

In order to use experimentally validated data for ligands of OR1A1, 3D structural data for a total of 106 chemical compounds (Supplementary Table 1)—53 agonists and 53 non-agonists—were applied to the analyses [6]. Three-dimensional files in the structure-data file (SDF) format for the 106 compounds were downloaded from the PubChem Compound Database at the National Center for Biotechnology Information (NCBI) and subjected to geometric optimization, molecular vibrational pattern analysis, docking simulation, and further study.

### Molecular vibration calculation and data formulation of OR1A1 ligands

To explore the site of the ligand that contacts the OR1A1 model, we adopted the revised corralled intensity of molecular vibrational frequency (CIMVF) of ligands as the molecular descriptor. Most of the analysis process followed previous reports [7,8], and a brief description of the procedure is as follows.

We utilized the CIMVF as the characteristic of each ligand molecule. Since the calculation of molecular vibrational frequencies requires a 3D structure of a given molecule, the geometric optimization of the ligand was carried out using the SDF file of each molecule. The theoretical 3D conformer SDF of each ligand molecule was modeled as a single low-energy conformation by using the Becke three-parameter Lee-Yang-Parr (B3LYP) density functional theory and the standard split-valence basis set 6-31G(d,p). Then, the result of geometric optimization was subjected to vibrational frequency calculation. The calculations of the geometric optimization and normal modes of molecular vibration were performed using the GAMESS program package [9]. When it was necessary to check the wavenumber of a substructure in a ligand molecule, we utilized MacMolPlt [10].

The wavenumbers of calculated molecular vibrations in a ligand molecule were sorted in increasing order and taken into each corral with a fixed step size (5 cm^-1^). As a molecular descriptor of a ligand, the intensity sum of each corral was arrayed in a one-dimensional vector containing 800 elements representing the wavenumber range of 0‒4,000 cm^-1^ [8].

### Feature selection by information gain

One of the challenges encountered when dealing with high-dimensional and sparse datasets such as CIMVFs of small molecules is that the number of important or informative features that must be grasped in order to understand the underlying mechanism of a particular phenomenon is very small. Feature selection using information gain (IG) is a process for reducing meaningless or less informative features.

Scoring with IG involves separately counting the occurrences of a feature in the agonist and non-agonist training examples, and then computing an equivalent function. The IG yielded from a dataset is given by the relative entropy between the prior and posterior probabilities [11]. When the information available is the presence of a feature and the corresponding class distribution, IG measures the amount of information about the class prediction in bits [12].

We adopted IG-based feature selection to identify the corrals of molecular vibrational frequency as the most informative features among the 800 elements for the classification of OR1A1 ligands into two types (agonists and non-agonists). Since most of the OR1A1 ligands have a small molecular weight, the number of corrals with molecular vibration is not large, so the density of the features will be quite sparse. We trained and tested the procedure by applying leave-one-out cross-validation to each of 106 ligands using the Weka machine learning package [13].

### Homology modeling of OR1A1

Since molecular docking requires a homology model of the target protein, it must be preceded by homology modeling of the corresponding protein. The amino acid sequence for OR1A1 (UniProt: Q9P1Q5) was obtained from UniProt KnowledgeBase (https://www.uniprot.org/). To choose the amino acid sequences of 7TMRs that have a higher BLAST score (bits) than 45 or a lower E-value than 1e-07, we analyzed the amino acid sequence of OR1A1 against the locally-built BLAST database of 7TMR amino acid sequences registered in the RCSB PDB. Based on their similarity results for OR1A1, four PDB entries of four 7TMRs were selected as the experimental templates for the homology modeling of OR1A1: human β2-adrenergic receptor (ADRA2A), human adenosine receptor (AdoRA2A), bovine rhodopsin (Rho), and turkey β1-adrenoceptor (ADRB1) PDB models.

In order to apply the activated structure of the receptor as the template, we adopted the PDB model of each receptor binding to an agonist or the corresponding G protein: 7BZ2 (PDB code for ADRA2A), 5G53 (AdoRA2A), 5TE3 (Rho), and 6H7J (ADRB1). The multiple sequence alignment (MSA) of these four PDB models was executed using T-Coffee (Rel. 11) in “slower and more accurate” mode [14]. We applied the MSA result to MODELLER (Rel. 9.25 [15]) and the application automatically combined these four templates to build the model for OR1A1 using information from multiple templates to build the three-dimensional (3D) structural model of OR1A1. After confirming the 3D model of OR1A1 obtained here with a Ramachandran plot, it was used for the subsequent docking experiment.

In addition to confirmation of the 3D model of OR1A1, Phobius was used to determine the terminal regions of the transmembrane α-helices of the OR [16].

### Molecular docking and scoring

In recent years, molecular docking has frequently been used as a practical computational methodology to predict the binding structure between a ligand and a receptor. There are several freely available programs for molecular docking analysis, such as smina [17]. Smina was created as a fork of AutoDock Vina optimized to support high-throughput and user-specified scoring. With reference to a report that smina has relatively high performance and is convenient to handle relative to several freely available docking programs, such as AutoDock4, AutoDock Vina, and idock [18], the subsequent docking and scoring experiments were performed using the smina program.

In order to simulate the binding of a ligand to a protein in a molecular docking tool, it is necessary to designate a spatial region within the protein to which the ligand can bind. We used AutoDockTools4 (ADT4), which accompanies AutoDock4, to prepare practical conditions for the 3D docking space in the modeled OR1A1 [19]. The binding site grid box was visually defined for the model of OR1A1 by employing the grid setting feature of ADT4.

To explore the structural conformation of each receptor-ligand set, smina was executed using the default parameters with the exception of the 3D coordinates of the search space so that the program outputs nine docking poses for each run. The 3D SDF files of each ligand were downloaded from the PubChem of NCBI since smina receives SDF files as input for the docking experiment with the corresponding receptor. The subsequent processes were carried out under the default conditions of smina.

## Results

### Molecular vibrational patterns of ligands and IG ranking

According to the results of the molecular vibrational frequency, 372 corrals of agonists and 366 corrals of non-agonists had vibrational intensities of 0, out of the 800 ones of CIMVF. The remaining corrals were regarded as features containing partial characteristics of the ligand with molecular vibration patterns. In the IG score calculations, only 11 out of over 400 features showed IG scores exceeding 0. Among them, nine wavenumbers that are physicochemically meaningful are shown in Table 1. Only informative features with IG scores larger than 0 are listed in the table.

**Fig. 1.**
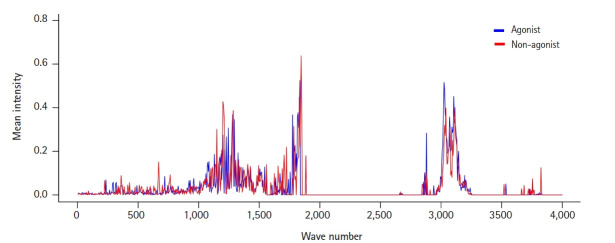
The mean intensities of CIMVF of the two ligand groups (agonists and non-agonists) for OR1A1. CIMVF, corralled intensity of molecular vibrational frequency; OR1A1, olfactory receptor 1A1.

To view the distribution and intensity of molecular vibrational frequencies as a whole, we also plotted the mean vibrational intensities of OR1A1 agonists and non-agonists according to their molecular vibrational frequency (Fig. 1).

There were three major areas of features with mean intensities greater than the mean value: around 1,200, 1,800, and 3,100 cm^-1^. Among them, the first region around 1,200 cm^-1^ seemed to be marked in a way that could distinguish agonists and non-agonists. This can also be confirmed in the features from the IG ranking data: F241 and F243 (Table 1). F230, the feature with the highest frequency, has a wavenumber only 50 cm^-1^ away from them.

The wavenumber 1,204.48 cm^-1^, belonging to F241, corresponds to the molecular vibrational frequency of the cyclohexanone ring in (+)-dihydrocarvone, and F230, which includes a wavenumber of 1,146.86 cm^-1^, is a representative feature of the molecular vibration of quinoline. Since the structure of the odorant molecules used in the analysis is relatively simple, a limitation is that the distinction of features corresponding to each substructure does not seem to have much meaning. However, molecular vibrational information of a specific substructure of a ligand is useful for identifying the sites where the receptor interacts. The relationship between agonism on the receptors and molecular vibrations of ligands needs further research.

### Homology model of OR1A1

**Fig. 2.**
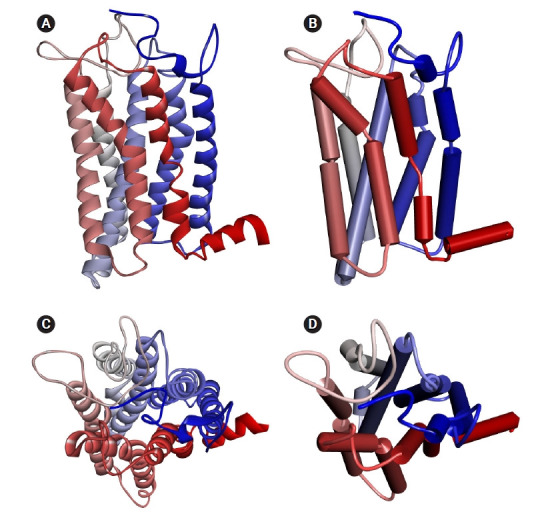
The homology model of human OR1A1 generated by MODELLER: (A) solid ribbon (side-view), (B) schematic (side-view), (C) solid ribbon (top-view), and (D) schematic (top-view). The colored transmembrane domains are shown in blue to red from the N-terminus to the C-terminus. The side-view and top-view stand for the view from the cell membrane parallel and outside the cell membrane, respectively. OR1A1, olfactory receptor 1A1.

The homology model of human OR1A1 generated by MODELLER with 4 PDB 7TMR models is shown in Fig. 2. The most conserved structure in ORs is the seven-helix bundle of transmembrane α-helical amino acids. In the figure, the color of each helix denotes the direction from the N-terminus (blue) to the C-terminus (red). In Fig. 2B and D, we can better recognize the structure of the receptor's binding pocket, which are formed by helices 3, 5, 6, and 7 with the schematic representation of the OR1A1 model.

**Fig. 3.**
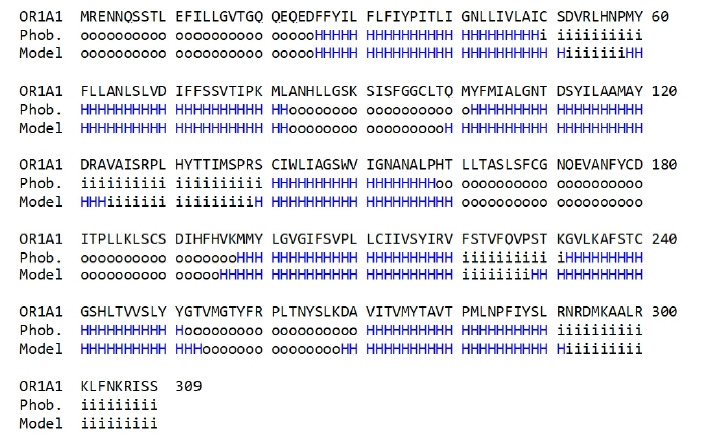
Sequence comparison of transmembrane (TM) regions between the olfactory receptor 1A1 (OR1A1) model (model) and those predicted with Phobius (Phob.). The TM amino acid sequence of the OR1A1 model was manually inspected. H: helical region (blue), o: outside of the cellular membrane, i: inside of the cellular membrane.

To compare the amino acid sequences in the terminal regions of the seven-transmembrane α-helices generated in the OR1A1 model to those produced by the sequence prediction tool, we used Phobius (Fig. 3).

As shown in Fig. 3, the transmembrane region of the homology model is aligned properly to that predicted using Phobius, supporting the validity of the 3D model of OR1A1.

### Docking dimensions between OR1A1 receptor model and ligands

In order to determine which site of each ligand binding to the previously generated OR1A1 model interacts with which site of the receptor, the distance between each ligand and the amino acid residue of the receptor located closest to it was determined. As shown in Table 2, the distance between the element group constituting the backbone of the ligand and the functional group of the nearest receptor amino acid residue was observed to be around 4 Å.

**Fig. 4.**
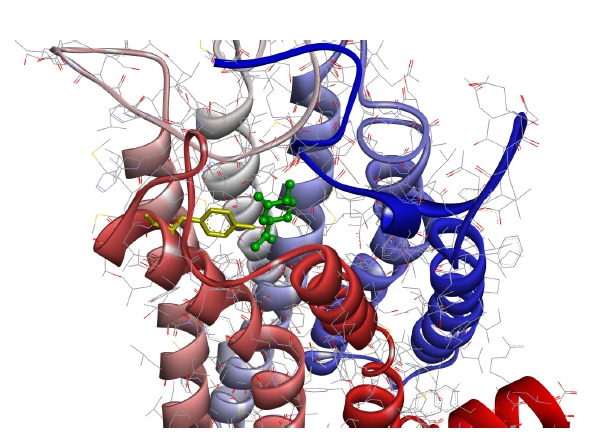
The conformation of (+)-dihydrocarvone (green, ball-and-stick) located nearest to Tyr251 residue (yellow, in the sixth transmembrane helix) of the OR1A1 model as viewed from the N-terminal side of the receptor. The colored transmembrane domains are shown in blue to red from the N-terminus to the C-terminus. OR1A1, olfactory receptor 1A1.

Fig. 4 shows the case with the minimum energy among the docking conformations with (+)-dihydrocarvone, which showed the shortest distance to the OR1A1 model.

As can be seen in the figure, (+)-dihydrocarvone, the ligand of OR1A1, and the Tyr251 residue of the OR1A1 model are not even 4 Å apart, and there are no amino acid residues of the OR close enough to bind the ligand around it.

## Discussion

In animal systems, ORs are the first proteins to detect signals from outside, and the mechanism of OR activation by ligands has not yet been elucidated in detail. It is known that odorant molecules bind to specific receptors through conventional molecular interactions, causing a conformational change in the receptor that initiates intracellular signals. However, this hypothesis was unable to distinguish or predict the properties of odorant molecules because a significant number of odorant molecules bind to a single OR. Therefore, it was not possible to design odorant molecules in consideration of the receptor’s characteristics [20].

If the ligand of a 7TMR has a large and complex structure, such as angiotensin with a molecular weight of 1,000 Da or more, it is very difficult to predict the mechanism of binding between the receptor and the ligand. In contrast, if the ligand has a small molecular weight and a chemically simple structure like the ligand of the OR1A1 receptor, it would be possible to track the process by which the ligand acts, binds to, and activates the receptor.

When electron tunneling was first described, it was exclusively the purview of physicists [21], but it is now also a very important theme in chemistry and biology. Recently, efforts have been made to interpret similar bitter almond odors between hydrogen cyanide, benzaldehyde, and nitrobenzene using the emission spectra of electron tunneling [22]. Studies of electron transfer in proteins such as electron hopping in polypeptides, electron transfer in peptides such as amino acid relays, and light harvesting systems in photosynthesis have been reported several times [23]. Quantum coherence must also be considered in relation to the Fenna-Matthews-Olson light-harvesting complex [24].

It is known that oxidoreductase proteins mediate tunneling of electrons at rates far faster than the substrate redox reactions they support. Electrons can travel up to 14 Å between redox centers through the protein medium [25]. Electron tunneling for a distance longer than 14 Å is possible through interventions such as cofactors.

The average distance between the molecular backbones of six ligands (Fig. 4) and the nearest amino acid residue of OR1A1 was 3.660 Å (Table 2). It has been reported that (+)-dihydrocarvone, with a molecular weight of 152.23 Da, is an agonist of OR1A1 [26]. The calculated distance between the oxygen atom of (+)-dihydrocarvone and the nearest oxygen of the Tyr251 residue of OR1A1 model was 3.260 Å in the docking simulation model (Fig. 4). Therefore, the distance between the OR and the ligand is not an obstacle to electron transfer.

**Fig. 5.**
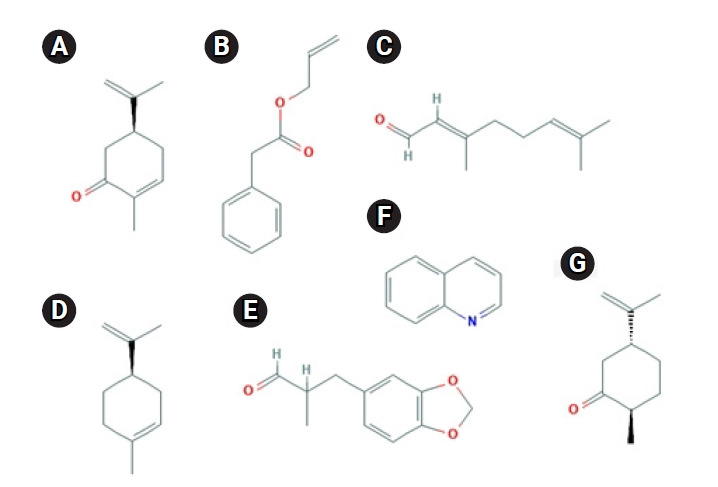
The chemical structures of seven agonists of OR1A1 [27,28]: (A) (+)-carvone, (B) allyl phenyl acetate, (C) citral, (D) D-limonene, (E) helional, (F) quinoline, and (G) (+)-dihydrocarvone. OR1A1, olfactory receptor 1A1.

Even if the odorant molecule interacts with another amino acid residue of other structures in OR1A1, the context of the above point is not expected to change significantly. This is because, as shown in Fig. 5 [27,28], most of the ligands of OR1A1 do not have a reactive group that would be capable of sending and receiving protons. In fact, odorant molecules do not change chemically when they bind to ORs.

The fact that such a chemically very simple ligand changes the structure of the receptor makes it undeniable that there is something very minute between them. It is thought that it would be impossible for odorant molecules to exert a strong enough influence to change the structure of a relatively large OR without any kind of physicochemical transportation. For example, if electron flow occurs, this phenomenon is likely to affect a salt bridge, such as an ionic lock [29], eventually changing the structure of the receptor. In this case, the conformational shift of TM6, a member of the ionic lock, reaches about 5 Å [30]. As an explanation for this, vibration-assisted tunneling can be proposed [31]. Once the electrons of the ligand are transferred in any way to the amino acid residues of the receptor, then there arises a possibility of affecting the ionic lock, either through single-step tunneling or a multi-step hopping process.

Research on the activation mechanism of ORs has been steadily progressing, but no model can fully explain this phenomenon. In particular, regarding the specific process through which receptors are activated by odorant molecules, whether the chemical structure of the molecule is the main factor or whether activation is due to the vibration of the molecule remains unclear. However, this inconsistency can be viewed as involving phenomena that are complementary to each other rather than mutually inconsistent possibilities. This is because the phenomenon through which the receptor recognizes and binds the ligand and the phenomenon through which the receptor is activated by the bound ligand may be different processes. More scrupulous and detailed follow-up studies are needed to clarify this issue.

## Figures and Tables

**Table 1.** The features with high frequencies and scores in the information gain (IG) analysis

IG feature No.	Wave number (cm^-1^)	Frequency
F601	3,000‒3,005	106
F286	1,425‒1,430	106
F230	1,145‒1,430	106
F108	535‒540	106
F764	3,815‒3,820	100
F241	1,200‒1,205	99
F243	1,210‒1,215	95
F368	1,835‒1,840	48
F347	1,730‒1,735	48

**Table 2.** The shortest distance between each ligand and the nearest amino acid residue of the OR1A1 model

Name	CAS No.	CID	Distance (Å)	B.A.
(+)-Carvone	2244-16-8	16724	4.132	‒6.4
Allyl phenyl acetate	1797-74-6	15717	3.574	‒6.2
Citral	5392-40-5	638011	3.468	‒6.0
D-Limonene	5989-27-5	440917	3.489	‒6.0
Helional	1205-17-0	64805	3.542	‒6.5
Quinoline	91-22-5	7047	3.755	‒6.1

OR1A1, olfactory receptor 1A1; B.A., binding affinity.

## Supplementary Materials

Supplementary Table 1. List of OR1A1 ligands
