# Supplementary material for: Implications of the simple chemical structure of the odorant molecules interacting with the olfactory receptor 1A1
Source: Genomics Inform. 2021 Jun 30;19(2):e18. doi: 10.5808/gi.21033 (PMC8261270; doi:10.5808/gi.21033)
Supplement: Supplementary Table 1. — List of OR1A1 ligands [file gi-21033suppl1.pdf]

Table S1. List of OR1A1 ligands.

Agonist	CAS	CID	Non-agonist	CAS	CID
(-)-carveol mix.	99-48-9	7438	(-)-2-Phenylbutyric acid	938-79-4	785330
(-)-Menthone	14073-97-3	26447	(-)-Camphor	464-48-2	444294
(+)-Carvone	2244-16-8	16724	(+)-Fenchone	4695-62-9	1201521
(+)-Dihydrocarvone	5524-05-0	22227	(+)-α-Pinene	7785-70-8	82227
(+)-Menthone	3391-87-5	443159	(+/-)-cis-Rose-oxid	16409-43-1	27866
(4R,4S)-γ-Nonalactone	104-61-0	7710	(4R,4S)-γ-Octalactone	104-50-7	7704
(R)-(+)-citronellal	2385-77-5	75427	(5R,5S)-δ-Decalactone	705-86-2	12810
(R)-(+)-citronellol	1117-61-9	101977	(5R,5S)-δ-Dodecalactone	713-95-1	12844
(R)-(+)-Limonene	5989-27-5	440917	(E,Z)-2,4-Nonadienal	21661-99-4	6429282
(S)-(-)-citronellal	5949-05-3	443157	(Z)-(3)-Hexenyl acetate	3681-71-8	5363388
(S)-(-)-citronellol	7540-51-4	7793	(Z)-4-Heptenal	6728-31-0	5362814
(S)-(-)-Limonene	5989-54-8	439250	2-(Mercaptomethyl)thiophene	6258-63-5	80408
1-Decanol	112-30-1	8174	2-(sec-butyl)-3-methoxypyrazine	24168-70-5	520098
1-Heptanol	111-70-6	8129	2-Aminoacetophenone	551-93-9	11086
1-Octen-3-ol	3391-86-4	18827	2-bromohexanoic-acid	616-05-7	12013
2-Nonanone	821-55-6	13187	2-Methylbutyl acetate	624-41-9	12209
2-Octanone	111-13-7	8093	2-Methylbutyraldehyde	96-17-3	7284
2-Pentylpyridine	2294-76-0	16800	2-Pentanone	107-87-9	7895
2-Phenylethanethiol	4410-99-5	78126	2,3-dietyl-5-methylpyrazine	18138-04-0	28905
2,4-Nonanedione	6175-23-1	80314	2,4-Dimethylphenol	105-67-9	7771
2,4-Octanedione	14090-87-0	84192	4-Ethylphenol	123-07-9	31242
3-Heptanone	106-35-4	7802	4-Hydroxycoumarin	1076-38-6	54682930
3-Mercaptohexyl acetate	136954-20-6	518810	4-Methylacetophenone	122-00-9	8500
3-Methyl-2,4-nonanedione	113486-29-6	529481	Abhexon	698-10-2	61199
3-Octanone	106-68-3	246728	Acetal	105-57-7	7765
3-phenylpropylpropionate	122-74-7	61052	Allyl benzene	300-57-2	9309
4-Chromanone	491-37-2	68110	Alpha-farnesene	502-61-4	5281516
6-Methyl-2,4-heptanedione	3002-23-1	76354	androstenone	18339-16-7	6852393
Allyl heptanoate	142-19-8	8878	Anisole	100-66-3	7519
Allyl phenylacetate	1797-74-6	15717	Benzene	71-43-2	241
androstadienone	4075-07-4	92979	Cinnamyl alcohol	104-54-1	5315892
Benzophenone	119-61-9	3102	citronellic acid	502-47-6	10402

Table S1. List of OR1A1 ligands.

Benzyl acetate	140-11-4	8785	Cyclohexanone	108-94-1	7967
Bourgeonal	18127-01-0	64832	Decanoic acid	334-48-5	2969
Butylanthranilate	7756-96-9	24433	Dihydroeugenol	2785-87-7	17739
Cinnamaldehyde	14371-10-9	637511	Dimethyl disulfide	624-92-0	12232
Citral	5392-40-5	638011	Dimethyl trisulfide	3658-80-8	19310
Dihydrojasmone	1128-08-1	62378	Dipropyl disulfide	629-19-6	12377
E-4-decenal	65405-70-1	5702654	E,E-2,4-dodecadienal	21662-16-8	5367530
Ethyl caproate	123-66-0	31265	Ethyl caprate	110-38-3	8048
Ethyl cyclohexanecarboxylate	3289-28-9	18686	Ethyl caprylate	106-32-1	7799
Ethyl phenylacetate	101-97-3	7590	Ethyl cinnamate	103-36-6	637758
ethylenebrassylate	105-95-3	61014	Furaneol	3658-77-3	19309
Helional	1205-17-0	64805	Isobutyraldehyde	78-84-2	6561
Hydroxy-citronellal	107-75-5	7888	Methyl 2-methylbutyrate	868-57-5	13357
nerol	106-25-2	643820	Methyl cinnamate	103-26-4	637520
nerolidol	7212-44-4	5284507	methyl salicylate	119-36-8	4133
Nonanethiol	1455-21-6	15077	Methyl-pentanal	123-15-9	31245
Phenethyl acetate	103-45-7	7654	o-Cresol	95-48-7	335
Quinoline	91-22-5	7047	Skatole	83-34-1	6736
shoyupyrazine	15707-24-1	27458	Succinic acid	110-15-6	1110
trans-Anethole	104-46-1	637563	trimethyl-pyrazine	14667-55-1	26808
Z-7-decenal	21661-97-2	5362695	β-Ionone	14901-07-6	638014
